# Right-Wing Politicians Prefer the Emotional Left

**DOI:** 10.1371/journal.pone.0036552

**Published:** 2012-05-02

**Authors:** Nicole A. Thomas, Tobias Loetscher, Danielle Clode, Michael E. R. Nicholls

**Affiliations:** 1 School of Psychology, Flinders University, Adelaide, South Australia, Australia; 2 School of Humanities, Flinders University, Adelaide, South Australia, Australia; French National Centre for Scientific Research, France

## Abstract

Physiological research suggests that social attitudes, such as political beliefs, may be partly hard-wired in the brain. Conservatives have heightened sensitivity for detecting emotional faces and use emotion more effectively when campaigning. As the left face displays emotion more prominently, we examined 1538 official photographs of conservative and liberal politicians from Australia, Canada, the United Kingdom and the United States for an asymmetry in posing. Across nations, conservatives were more likely than liberals to display the left cheek. In contrast, liberals were more likely to face forward than were conservatives. Emotion is important in political campaigning and as portraits influence voting decisions, conservative politicians may intuitively display the left face to convey emotion to voters.

## Introduction

“There is only one party in the United States, the Property Party…and it has two right wings: Republican and Democrat” [1]. Although Gore Vidal may bemoan the conformity of conservative and liberal politics, both physiological and behavioural research suggests otherwise. While political choice is undoubtedly influenced by an individual's social experience, it has also become increasingly apparent that biological and neurological processes play an integral role in shaping our attitudes and beliefs, such as those relating to politics [2], [3].

The importance of physiology in determining political affiliation is clearly demonstrated by genetic studies. Alford, Funk, and Hibbing [4] found that, among twins, although socialization was an important factor in determining political ideology, genetic influences were twice as influential as the environment. This suggests that political attitudes themselves are highly influenced by genetics [4]–[6].These studies demonstrate that the heritability of political ideologies is not the result of socialization alone, but is best explained by the combination of genetics and environmental influences [7].

The genetic transmission of political ideology may be reflected in specific anatomical regions of the brain. The grey matter of the anterior cingulate cortex is larger in self-reported liberals than conservatives [8]. This part of the brain, which is important for conflict resolution, also produces larger event-related potentials in liberals [9]. Conservatives, in contrast, show increased grey matter volume in the right amygdala [8]. As the amygdala is important for emotional control, it is possible that these underlying brain differences lead conservatives and liberals to differ in their perception, expression and experience of emotion.

It has been shown that conservatives have a heightened sensitivity for detecting emotional faces [10] and for feeling disgust [11]. In addition, when given cleanliness reminders, individuals exhibit more conservative political attitudes, suggesting a link between thoughts of physical purity and political attitudes [12]. These findings suggest that self-reported conservatives and liberals differ in their experience of emotion and provide potential reasons for which conservatives are said to use emotion more effectively in their political campaigns [13].

Although the precise role of emotions in political campaigning has yet to be elucidated, it is clear that emotions are important in appealing to voters. We might be inclined to believe that political campaign advertisements serve to inform citizens; however research has indicated this is not necessarily the case [14]. As opposed to educating voters, political campaigns appear to primarily persuade voters [14] by appealing to their emotions, which subsequently influences their voting decisions [15]. Brader [15] demonstrated that by drawing on our emotions, including fear and enthusiasm, the effectiveness of political advertisements was increased.

The overt manipulation of specific emotions is only one example of how political campaigns could make use of emotion. The way in which the left and right sides of the face are portrayed also influences the intensity and perception of emotion [16]. The lateralization of emotion remains a controversial topic, as two competing theories have been supported. The right-hemisphere hypothesis suggests that the more dominant role of the right cerebral hemisphere in emotion processing as well as its control over the left side of the body leads the left half of the face to be more emotionally expressive [16]. Alternatively, the valence hypothesis proposes that the right hemisphere is only dominant for negative emotions, whereas positive emotions are processed by the left hemisphere [17]. Although the right-hemisphere hypothesis has received more consistent support [18], numerous studies have also provided support for the valence hypothesis [19], with some suggestion that these hypotheses are likely not mutually exclusive [20].

Interestingly, the left cheek is often displayed more prominently than the right cheek in portraits and photographs [21], [22]. This leftward bias is strongest when the model wants to display emotion, but is eliminated when concealing emotion [23]. A number of studies have demonstrated that emotions are rated as more expressive when they are displayed on the left side of the face [22] and individuals who are more emotionally expressive are more likely to present the left cheek when posing for a portrait [24]. Portraits featuring the left face are judged as more emotional [25] and the leftward posing bias also appears to be stronger among females [24]. The preference for turning the left cheek in portraits appears to be the result of right hemisphere activation [22]–[25].

As activity in the amygdala increases when individuals view the face of a hypothetical candidate for whom they would vote [26], it was of interest to examine whether political ideology influenced the emotional content of the official photographs of members of Parliament and Congress. It has recently been shown that individuals who are less informed on political issues are more likely to vote based solely on the appearance of politicians [27], making it even more appealing to examine photographs of politicians. If conservatives are more predisposed to express and perceive emotion, they should be more likely to present the emotional left cheek more prominently in portraits. Consistent with prior research [24], it was also expected that females would be more likely to show the left cheek.

## Results

**Figure 1:**
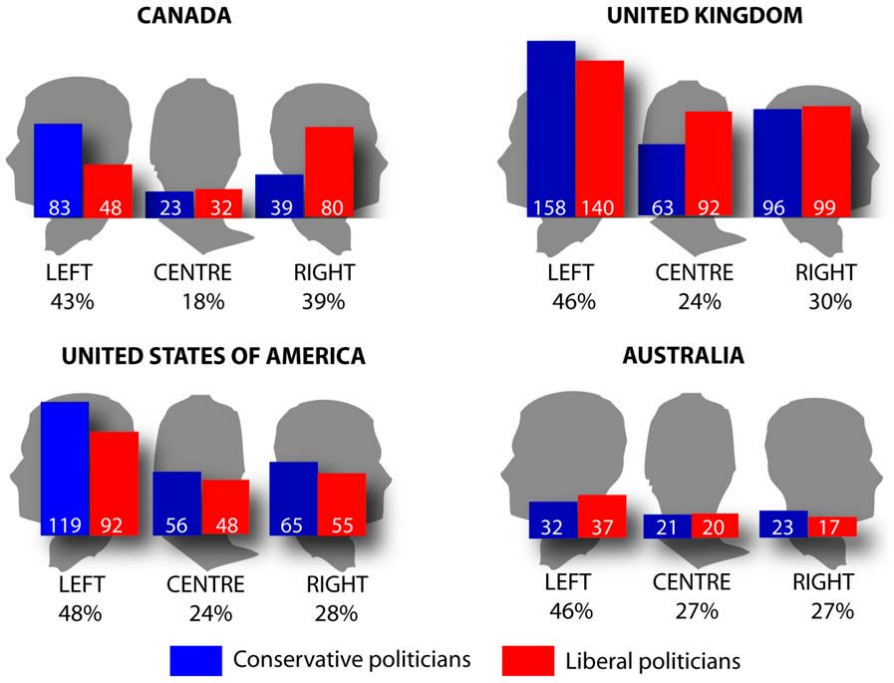
Frequency of left, centre and right poses for conservative and liberal politicians from Canada, the UK, USA and Australia.

Forward facing poses (N = 355) were omitted from the first analysis as it was primarily of interest to compare left and right facing images. A hierarchical loglinear analysis assessed the effect of sex (male, female), political position (liberal, conservative), country (Canada, USA, UK, Australia) and posing bias (left, right). To control for multiple comparisons a more conservative p value of 0.01 was used to determine significance. There was a significant interaction between country, political position and posing bias (χ^2^ (3, *N* = 1183) = 18.667, *p*<.001; see Figure 1). The interaction of bias and country was then examined separately for conservative and liberal political positions. For conservative politicians the interaction was not significant, χ^2^ (3, *N* = 615) = 2.036, *p* = .565, indicating that a similar pattern was observed for all countries. In each nation leftward posing biases occurred more often than rightward ones. For liberal politicians the interaction was significant, χ^2^ (3, *N* = 568) = 24.414, *p*<.001. Although liberal politicians showed a reduced leftward posing bias, Canadian left-wing politicians showed a rightward posing bias (63%; see Table 1).

**Table 1:** Number of cases included in each condition.

			Posing Bias		
Country	Sex	Political Position	Left	Forward	Right
Australia	Male	Conservative	27	14	21
		Liberal	27	11	13
	Female	Conservative	5	7	2
		Liberal	10	9	4
Canada	Male	Conservative	74	13	35
		Liberal	37	19	60
	Female	Conservative	9	10	4
		Liberal	11	13	20
United Kingdom	Male	Conservative	140	46	80
		Liberal	108	56	74
	Female	Conservative	34	36	25
		Liberal	18	17	16
United States	Male	Conservative	109	47	60
		Liberal	70	33	44
	Female	Conservative	10	9	5
		Liberal	22	15	11

A 2×2 chi-square was carried out to examine the interaction of sex (male, female) and bias (left, right). The interaction was not significant, χ^2^ (1, *N* = 1183) = .487, *p* = .485, demonstrating that sex did not account for any of the differences in directional posing bias. As sex differences have been previously observed, it was of interest to examine whether males and females differed in the forward facing poses. A 2×2 chi-square was carried out to examine the interaction of sex (male, female) and bias (left+right, forward). The interaction was significant, χ^2^ (1, *N* = 1538) = 38.428, *p*<.001. Although 67% of forward facing portraits were of males, a higher percentage of female photographs (36%) than male photographs (20%) showed a forward facing pose.

A 2×4 chi-square was used to examine the interaction of country (USA, Canada, Australia, United Kingdom) and bias (left, right). The interaction was not significant, χ^2^ (1, *N* = 1183) = 8.482, *p* = .037. A 2×2 chi-square analysis was also employed to examine the interaction of political position (liberal, conservative) and bias (left, right). The interaction was significant, χ^2^ (1, *N* = 1183) = 7.733, *p* = .005. Although leftward poses occurred more often for both political parties, conservative politicians were more likely to exhibit leftward poses (64%) than were liberal politicians (54%). Lastly, a chi-square analysis was used to examine the main effect of posing bias. The result was significant, χ^2^ (1, *N* = 1183) = 46.682, *p*<.001, demonstrating that leftward poses (60%) occurred more frequently than rightward poses (40%).

## Discussion

Overall, politicians were more likely to display the left cheek in their official photographs, consistent with prior reports of a leftward posing bias in portraiture [21], [22]. Interestingly, conservative politicians were significantly more likely to display the left cheek bias than were liberal politicians. Results indicated there was an interaction between the variables of posing bias, country and political position. Examination of liberal and conservative political positions separately revealed that the pattern of findings was consistent across countries for conservative politicians, with a leftward bias being more common in all instances. For liberal politicians, a reduced leftward bias was seen.

It is of interest to note that liberal politicians from Canada demonstrated a rightward posing bias. This finding also accounts for the overall decreased leftward bias observed among Canadian politicians. Prior research has shown that the right cheek is more likely to be exposed when individuals are asked to pose for a scientific portrait or when concealing emotion [23]. This suggests that while liberal politicians were less likely to employ emotion in their photographs, Canadian liberals were in fact attempting to conceal emotion by displaying the right cheek.

Comparisons were also carried out between directional poses and those that were forward facing. Directional poses occurred more often, with the number of forward facing images being similar across countries. Liberals were more likely face forward than were conservatives. This could reflect a desire by liberal politicians to appear emotionally neutral as opposed to making use of emotion in their official photographs.

Interestingly, females showed a stronger tendency to face forward than males. This contrasts with prior research showing that females are more likely to present the left cheek [22], [24]. It has been shown that gender stereotypes can significantly influence public perception of female politicians [29]–[32]. This suggests that female politicians intuitively face forward and are less likely to make use of emotion in official portraits to avoid being seen as emotional.

Conservative politicians are more likely than liberals to have portraits featuring the left face. It is possible that the left cheek bias could reflect a desire to conform to status quo by presenting the cheek that is more commonly shown in paintings and portraiture [21]–[25]. As such, conservative politicians might be likely to exhibit a “traditional” pose in their portraits. Although such a possibility cannot be excluded, prior research suggests that the desire to display or conceal emotion influences which cheek is displayed more so than one pose necessarily being more traditional [21]–[23]. Given the predisposition of conservatives to express and use emotion [8], [10]–[13], the preference to show the left cheek would allow conservatives to communicate emotions to voters through their portrait. The leftward bias is likely intuitive and driven by right hemisphere specialisation for emotion processing [22], [23].

There is increasing evidence that research combining biology, political science, and psychology is crucial for understanding the underlying role of the brain in political attitudes and ideologies as they relate to actual behaviour, such as voting decisions [2], [3]. The link between social behaviours, such as voting, and an individual's genetics appears to operate through a number of stages, one of which is proposed to be emotional processing [3]. Such suggestions, when combined with research showing that emotion is an important component in political campaigns [13], [15], [33], demonstrates the importance of understanding the various ways in which emotion can be communicated to the voting public.

Rather than showing that conservative and liberal politics are the same, this research highlights differences between the two ideologies, which appear to be both rooted in the brain [8], [9], [26] and highly heritable [4]–[7]. The current findings suggest that conservatives make better use of emotion than liberals by presenting the more emotional left cheek. Future research should examine more directly whether the left cheek bias indeed influences the use of emotion in political campaigns. -Emotion is an important component in political campaigns [13], [15], [33] and portraits play a significant role in voter decisions [26], [27]. Given that voters are persuaded by political advertisements [15], less informed voters, who are more likely to vote based on appearance [27], might vote for politicians with more emotional official photographs. The cheek that politicians turn may serve as an important indicator of the role emotion plays in their political ideology and how they will tackle ongoing social issues.

## Methods

Photographic portraits (N = 1538) were collected for Australian, British, and Canadian members of parliament and American congressmen and congresswomen. Official photographs, found on national government websites or individual member websites (http://www.aph.gov.au/; http://www.parl.gc.ca; http://www.parliament.uk; http://www.house.gov) between 10 March 2011 and 22 April 2011 were examined.

Images were coded as having either the left or the right cheek more visible by measuring each side of the face from the centre of the nose. The widths of the two sides of the face were compared, with the longer side being the more prominent one. Images in which the two sides differed by less than five percent were coded as forward facing. Each member's name, political party, state and nationality were also recorded.

In an effort to include all parties, descriptions of political ideology (e.g., social democracy, liberal conservatism, nationalism, green politics) were determined from publicly available information online. Political ideology was classified as either left or right of the centre on the left-right political spectrum. Classification accounted for position along the dimension of radicalism (i.e., economic and social issues) and the dimension of libertarian (individualist) compared to authoritarian (communitarian) [28]. For example, the political ideology of the Australian Labour party is in line with that of a social democracy and is therefore left of centre. The Liberal Party of Australia has an ideology in line with liberal conservatism and is therefore right of centre. For ease of comprehension regarding left- and right- wing politics and left and right sides of the face, left-wing politicians are referred to as liberal and right-wing politicians are referred to as conservative.
